# Quantitative studies of translymphnodal passage of tumour cells naturally disseminated from a non immunogenic murine squamous carcinoma.

**DOI:** 10.1038/bjc.1975.4

**Published:** 1975-01

**Authors:** H. B. Hewitt, E. Blake

## Abstract

A squamous cell carcinoma of spontaneous orgin in a WHT/Ht mouse was used to study the frequency with which the regional axillary lymph nodes draining subcutaneous or intradermal tumours gave rise to tumours after their isogeneic transplantation as whole nodes. This frequency (similar to 40%) was found not to vary significantly with the size or duration of the tumour drained and not to be increased by coincident infective, traumatic or antigenic stimuli acting at the tumour site or in adjacent tissue. Because tumour growth occurred in only 2/55 (4%) nodes which were left in situ in mice whose tumours were radically excised, it was concluded that tumour forming node transplants reflected a small and limited content (estimated to be about 13) of transnodally passing tumour cells destined to pass on to the blood; separate experiments showed that tumour cells reaching the blood survived for only a few hours. Nodes from tumour-excised mice gave rise to tumours as frequently when autografted as when isografted to mice with no previous expose to the tumour. A review of the finding reported here and of previous quantitative data for this system enabled us to exclude any implication of anti-tumour immunity from our interpretation of the results of the experiments.


*Br. J. Cancer* (1975) **31**, 25

### QUANTITATIVE STUDIES OF TRANSLYMPHNODAL PASSAGE OF TUMOUR CELLS NATURALLY DISSEMINATED FROM A NON-IMMUNOGENIC MURINE SQUAMOUS CARCINOMA

H. B. HEWITT AND E. BLAKE

*From the C. R. C. Gray Laboratory, Mount Vernon Hospital, Northwood, Middlesex HA6 2RN, England*

Received 26 July 1974. Accepted 19 August 1974

**Summary.—A squamous cell carcinoma of spontaneous origin in a WHT/Ht mouse was used to study the frequency with which the regional axillary lymph nodes draining subcutaneous or intradermal tumours gave rise to tumours after their isogeneic transplantation as whole nodes. This frequency (~40%) was found not to vary significantly with the size or duration of the tumour drained and not to be increased by coincident infective, traumatic or antigenic stimuli acting at the tumour site or in adjacent tissue. Because tumour growth occurred in only 2/55 (4%) nodes which were left *in situ* in mice whose tumours were radically excised, it was concluded that tumour forming node transplants reflected a small and limited content (estimated to be about 13) of transnodally passing tumour cells destined to pass on to the blood; separate experiments showed that tumour cells reaching the blood survived for only a few hours. Nodes from tumour-excised mice gave rise to tumours as frequently when autografted as when isografted to mice with no previous exposure to the tumour. A review of the findings reported here and of previous quantitative data for this system enabled us to exclude any implication of anti-tumour immunity from our interpretation of the results of the experiments.**

SQUAMOUS cell carcinoma is one of the commonest types of clinical cancer and lymph nodal metastasis is the most frequent, and commonly the only, route of its dissemination. For example, of 2044 cases of squamous carcinoma of the upper respiratory and digestive tracts seen at the M. D. Anderson Hospital between 1948 and 1965, 57% had clinical evidence of nodal metastasis at the time of first presentation (Lindberg, 1972). It is curious that a manifestation of such clinical prominence appears to have stimulated very little continuing experimental study of its pathogenesis using appropriate animal tumour systems.

For an animal tumour system to be an acceptable model of a particular form of human malignancy, regard has to be paid not only to histological type but also to the avoidance of artefactual immunological features associated with the origin of the animal tumour. This is especially so when the investigations involve an immunologically significant organ such as the lymph node. Allografted tumours clearly entail irrelevant transplantation immunity. Tumours induced by powerful chemical carcinogens usually display antigenicity when isogeneically transplanted; several workers have used a single method to compare spontaneous and chemically induced tumours in respect of their relative capacities to evoke a rejection response on isogeneic transplantation (Prehn and Main, 1957; Marchant, 1968; Klein, 1970); in all these investigations, nearly all chemically induced tumours were found to be antigenic whereas tumours of spontaneous origin were rarely so, or not at all.

With these considerations in mind, we have reviewed abstracts of all the articles listed under " lymphnodal metastasis " in *Excerpta Medica* (Section 16—*Cancer*) for the period 1967–72. Of the 347 articles, only 16 (5%) were reports of experimental studies. However, all but 2 of the animal tumours used were either allografts or grafts of chemically induced tumours, the possible exceptions being an ascites tumour and a lymphoma.

The experiments to be described were carried out using a squamous cell carcinoma of spontaneous origin and transplanted within the inbred subline of mice in which it arose.

We present first a summary of the results of transplantation assays of cells of this tumour injected subcutaneously (s.c.) or intravenously (i.v.). Only a small number of cells was required for successful s.c. transplantation and this number was not increased by preliminary " immunization " of the recipients with lethally irradiated cells of the same tumour. On the other hand, cell numbers at least 100 times larger failed to give rise to disease after i.v. injection; related experiments involving transplantation of whole lungs of i.v. injected mice suggested that tumour cells entering the circulation are destroyed within a few hours. Following this preliminary account of our assay data, we describe experiments in which the regional nodes draining s.c. or intradermal (i.d.) tumours were excised at various stages of tumour growth and transplanted s.c. as whole nodes to isogeneic mice. About 40% of such transplanted nodes gave rise to tumours and this proportion did not vary with the size or duration of the tumour drained; also, it was not influenced by the coincident application of infective, traumatic or antigenic stimuli to the region of the tumour. Equivalent nodes left *in situ* in tumour-excised mice observed for long periods very rarely gave rise to tumours. We conclude that tumour cells are continually streaming through the regional node without either accumulating in it or establishing metastatic growth. We suggest that cells pass through the node into the venous blood, where they are destroyed by the same mechanism as that responsible for the destruction of i.v. injected tumour cells. We draw attention to numerous features of our experiments which strongly discourage any immunological interpretation of our findings.

### MATERIALS AND METHODS

All handling of biological materials was done under fully aseptic conditions.

*Mice.*—Female mice of the inbred albino strain WHT/Ht were used in all experiments. They were bred in this laboratory and were aged 2–3 months when used. The method of breeding was such that the mice in any experiment were derived from multiple sublines.

*Tumour.*—The squamous cell carcinoma arose spontaneously in the skin of a female of the WHT colony providing the mice used. It was maintained by serial subcutaneous transplantation and the present studies were carried out using tumours from the 188th to 290th serial passages. The only change observed in the tumour since its initiation has been loss of keratin formation; the characteristic histology of a poorly differentiated squamous cell carcinoma has been preserved. End-point dilution assays of subcutaneously injected cells done at intervals during its history have given $TD_{50}$ values in the range 7–25 cells (mean 14 cells); that is, an average inoculum of 14 cells gives rise to tumours in 50% of the injected sites. The distribution of " take " frequency in relation to the inoculum size has not departed significantly from a Poisson distribution (Porter, Hewitt and Blake, 1973).

*Preparation and injection of tumour cell suspensions.*—Single-cell suspensions were prepared from tryptic digests of tumour mince by a method described previously (Hewitt, 1966). Counted cell suspensions were diluted to contain the required inoculum in 0·2 ml for i.v. injections, 0·1 ml for s.c. injections, and 0·015 ml for i.d. injections. Subcutaneous or i.d. injections were sited in the anterior axillary line just caudal to the costal margin; it had been established previously by the injection of patent blue that this site lies within a wide area of tissue draining to the axillary node.

*Transplantation of lung tissue.*—Both lungs were removed from mice at intervals after i.v. injection of tumour cells, finely minced and injected subcutaneously. The soft palpable masses produced by the inocula were quite distinct from the hard, progressively growing tumours to which a proportion of such inocula gave rise.

*Excision and transplantation of lymph nodes.*—Mice usually have a single axillary node situated beneath the pectoral muscle adjacent to the axillary vessels; the nearest other node lies on the biceps muscle of the forelimb (Hummel, Richardson and Fekete, 1966). Nodes to be excised post mortem were readily exposed by reflection of the cut pectoralis muscle. Axillary nodes were removed from living mice under ether anaesthesia: a 7 mm incision was made in line with the lower border of the free part of the pectoralis muscle, which was retracted in a cephalic direction; the node was exposed by blunt dissection until it could be cradled in forceps; deep connections were severed also by blunt dissection and traction; axillary haemorrhage was rare and operated animals developed no sign of damage to the axillary vessels or nerves. One or 2 incisions were made through the capsule of excised nodes immediately before their transplantation into deep pouches made in the subcutaneous tissue of the loin by opening sharp-pointed scissors inserted through a 3 mm skin incision, which was later closed by a metal clip. Nodes were autotransplanted to the contralateral loin.

*Excision of tumours.*—Intradermal tumours were excised under ether anaesthesia by removal of an ellipse of skin providing for a minimum clearance of 2 mm round the tumour. Recurrence at the site of excision was rare. The use of intradermal tumours, where excision is required, provides for a much more humane procedure than that most commonly practised—disabling amputation of a tumour-bearing hindlimb.

*Observation times.*—Mice which had received node transplants or which had had tumours excised were observed for tumour development for at least 60 days (over 100 days in some experiments). The maximum time for development of tumours from transplanted nodes was 33 days in the present experiments; a similar maximum time was observed for the development of tumours from minimal inocula of subcutaneously injected tumour cells.

*Statistical test.*—Frequencies of tumour formation from transplanted nodes were compared for significant differences by construction of a " four-fold " table from which $\chi^2$ values were calculated. Values of $P$ were taken for one degree of freedom.

### RESULTS

### 1. *Quantitative transplantation of tumour cells by the subcutaneous or intravenous route*

Thirteen subcutaneous assays of the cells made at intervals during the 8-year history of the tumour have given $TD_{50}$ values in the narrow range 7–25 cells (log mean, 14 cells). The Figure, comprising the data of 5 recent assays, shows the distribution of median latent periods for tumours arising in groups of equally injected sites in relation to the logarithm of the number of cells injected expressed as a factor of the $TD_{50}$ value obtained in the relevant assay. The regression line was drawn by the method of least squares. It will be noted that the latent period for development of just palpable tumours at the $TD_{50}$ level is 18 days.

An assay of cells injected intravenously gave a $TD_{50}$ value of 2000 cells; that is, no disease ensued in half the mice receiving this number of cells. Comparison of this i.v. $TD_{50}$ with the mean s.c. $TD_{50}$ (14 cells) shows that over 99% of potentially clonogenic cells are destroyed after they enter the circulation. The results of the following experiment suggest that these cells are destroyed within a a few hours of injection.

### 2. *Rate of disappearance of tumour cells from the lungs of mice injected i.v.*

In 2 separate experiments 200 tumour cells were injected i.v. into a number of normal isogeneic mice. At intervals after injection, groups of 5–10 mice were killed and their minced lungs were transplanted

FIG.—The relationship between the mean number of cells of WHT squamous cell carcinoma " D " injected subcutaneously (expressed as a factor of the $TD_{50}$ in the relevant assay), and the mean latent period for appearance of just palpable tumours. The regression line was drawn through the points by the method of least squares. The mean latent period at the $TD_{50}$ level is 18 days.

s.c. as described previously. The proportions of intravenously injected mice whose transplanted lungs gave rise to tumours at specified times after injection are recorded in Table I. Additionally, 10 injected mice were observed for 77 days without interference; none developed any disease. Since a substantial proportion of the injected cells appear to have lost their growth potential within 6 h of their first exposure to the host, it is clear that induced host immune influences cannot have been responsible for their destruction.

TABLE I.—*Rate of Loss of Tumour Cells from Lungs of Mice Injected i.v. with 200 Cells*

| Time after cells i.v. | Lung sets transplanted | Lung sets giving tumours |
|---|---|---|
| 5 min | 10 | 9 |
| 60–90 min | 5 | 4 |
| 5–6 h | 5 | 1 |
| 20–24 h | 10 | 0 |
| 48 h | 5 | 0 |

Of 10 mice left intact after injection, none developed disease in 77 days of observation.

3. *Effect on $TD_{50}$ of pretreatment of mice with lethally irradiated cells of the same tumour*

At the completion of the experiments reported here, viable tumour cells were assayed subcutaneously in mice which had received intraperitoneal inocula of $1{\cdot}2 \times 10^6$ lethally irradiated (LI) cells 15 days previously and $0{\cdot}3 \times 10^6$ LI cells 8 days previously. The $TD_{50}$ in the putatively " immunized " mice was 12 cells. An assay in similarly pretreated mice done 5 years previously yielded a $TD_{50}$ of 10 cells. Neither of these $TD_{50}$ values is significantly different from the mean $TD_{50}$ (14 cells) for assays done in untreated mice.

This failure to increase quantitatively the resistance of mice to viable cell inocula by standard " immunization " procedures is strong evidence against this tumour's being potentially antigenic in the mice used. Further evidence of non-antigenicity appears in the results of experiments described below. We should add that failure to increase the $TD_{50}$ by pretreatment of the recipients with LI cells has been our invariable experience with the many tumours of spontaneous origin for which attempts have been made.

4. *Effect of laterality of tumour on frequency of tumour formation by transplanted regional nodes*

In 6 separate experiments set up for various purposes, mice received equal inocula of tumour cells on the 2 sides, and the 2 axillary nodes were subsequently transplanted separately but at the same time after injection. The overall incidences of tumour formation from the transplants were as follows: right side—43/142; left side—42/142. Thus, there was no asymmetry in respect of the content of tumour cells in the regional node. This information provides an assurance that no anatomical peculiarity of the left side requires consideration in the interpretation of the many experiments in which tumours were implanted only on the left.

5. *Effect of tumour size at the time of node transplantation on the frequency of tumour formation by the transplant*

It is commonly and reasonably assumed that, for a given tumour, the chance of tumour cells having migrated to a regional node increases with tumour size. A logical presentation of this assumption is that this chance is positively correlated with some function of the tumour growth curve which integrates tumour cell population size and time. To explore this assumption, we have analysed as a whole the results of several separate experiments in which tumours were excised and weighed at the time nodes were transplanted for testing of their tumour cell content. The results of this study are given in Table II. It is to be noted (Group 1) that there were 2 instances of tumour formation by nodes draining tumour inoculum sites

TABLE II.—*Frequency of Tumour Formation by Transplanted Regional Nodes in Relation to Tumour Weight at the Time of Transplantation*

| Group | Tumour weight (mg) | No. of tumours | No. of +ve nodes (%) |
|---|---|---|---|
| 1 | Not palpable | 49 | 2 (4) |
| 2 | 20– 250 | 47 | 14 (30) |
| 3 | 251– 500 | 78 | 24 (31) |
| 4 | 501–1000 | 26 | 15 (58) |
| 5 | 1001–1500 | 12 | 6 (50) |
| 6 | 1501–2000 | 6 | 3 (50) |
| 7 | 2001–3000 | 2 | 1 (50) |
| Totals (excluding Group 1) | | 171 | 63 (37) |

in which no tumour could be detected; it may be added here that one mouse bearing a 3·0 g tumour was shown to have tumour cells in its contralateral, as well as its ipsilateral, node. It is of interest that there have been clinical analogies of both these unusual findings.

Table II indicates that the frequency of positive node transplants increases only very slightly with tumour size. The pooled frequency for tumours up to 500 mg (30%) is significantly less than the pooled frequency (54%) for all larger tumours ($P<0{\cdot}01$). However, the Poisson curve relating tumour incidence to inoculum size indicates that a rise from 30% to 50% in tumour incidence requires only a two-fold increase in inoculum size. Since the larger tumours have resided in their hosts for a considerably longer period than the smaller tumours, the data of Table II do not support a hypothesis that the tumour cell content of a regional node increases in proportion to some integral function of the size, and duration in the host, of the tumour which the node drains. Our conclusion from this analysis is that the number of tumour cells present in the regional node at any time during tumour growth is largely independent of the number of cells reaching it and is limited by some characteristic of the node tentatively described as its " holding capacity ". A more detailed exposition of this concept is given in the Discussion of this paper.

6. *Incidence of positive node transplants following inocula of tumour cells into subcutaneous air sacs*

It was conceivable that the local increase of hydrostatic pressure coincident with injection could drive tumour cells directly into the lymph node at that time. Such artefactual propulsion of cells had to be distinguished from the natural migration of cells to the node during the course of tumour growth.

An inoculum of $4 \times 10^4$ tumour cells was injected into a group of mice bearing preformed air sacs made by the s.c. injection of 3 ml of air (Hewitt, 1956); a control group received the same inoculum by ordinary s.c. injection. Between 17 and 21 days after injection, when the mice bore tumours of 5–10 mm diameter, pairs of mice (one from each group) had their regional nodes excised and transplanted. The incidence of positive nodes from the air sac mice (3/15) was not significantly less than that of nodes from the control mice (5/17), although injection into air sacs would protect against local hydrostatic pressure at the site of injection.

Further evidence against the artefactual condition postulated was given by our observation that the frequency of positive nodes was not influenced by a 100-fold difference in the size of the inoculum used to initiate tumours.

7. *Effect on frequency of positive nodes of the application of traumatic, bacterial or antigenic stimuli to the tumour region*

Experiments under this heading were instigated by the postulation that involvement of the node in functional reactivity to the various stimuli might influence its content of tumour cells.

In each experiment, 2 groups of mice received equal unilateral inocula of tumour cells; one of the groups received the complicating procedure, the other serving as a control; transplantation of nodes was carried out at various times during tumour growth but each transplant session included equal numbers of nodes from the 2 groups. The different procedures (A–E in Table III) were as follows: A—$10^4$ live *E. coli* were added to each inoculum of tumour cells; 8 tumours plated on blood agar between the 4th and 12th days after injection were found to be sterile, so that tissue infection was evidently controlled at any early stage. B—$2 \times 10^5$ $\beta$-haemolytic streptococci were injected at a separate site adjacent to the tumour 7 days after injection of tumour cells and 5–8 days before transplantation of nodes; no abscess arose in the infected sites. C—the injection sites, or tumours when they had arisen, were firmly massaged between finger and thumb every 2 days between the 4th and 12th days, nodes being transplanted from the 12th to 14th day. D—on the 8th day after injection of tumour cells, a deep cruciate incision was made through the greater part of the thickness of the tumours, nodes being transplanted 6 days later. E—$2 \times 10^5$ viable cells of a foreign (CBA) sarcoma were injected into the medial end of the pectoral muscle 48 h before the i.d. injection of carcinoma cells at a site at least 1 cm away; growth of the allograft was variable in different mice but was to about 5 mm in many by the time of node transplantation.

The results of these experiments (Table III) show that the frequency of positive nodes in any complicated group is not significantly different either from

TABLE III.—*Effect of Complicating Stimuli Applied to the Tumour Region on the Frequency of Tumour Formation (T.F.) by Transplanted Regional Nodes*

| Experiment | Added stimulus | T.F. nodes/nodes transplanted (%) Test mice | Control mice |
|---|---|---|---|
| A. | $10^4$ *E. coli* | 5/18 (28) | 6/18 (33) |
| B. | $2 \times 10^5$ streptococci | 7/28 (25) | 9/28 (32) |
| C. | massage | 7/30 (23) | 13/30 (43) |
| D. | incision | 11/28 (39) | 14/28 (50) |
| E. | allograft | 8/15 (53) | 7/13 (54) |
| | Total | — | 49/117 (42) |

that in its own control group or from the pooled value for the 5 control groups. It is estimated from the Poisson curve for data from subcutaneous assays that, had any of the complicating procedures increased the average number of tumour cells per node by a factor of only 3, a significant difference would have been expected in any of the experiments done.

It is concluded that the number of tumour cells in a node at the time of transplantation is not grossly altered by coincident influences on its functional state. The results of the allograft experiment (E) indicate that participation of a node in the process of allograft rejection does not reduce its capacity to hold isografted tumour cells and does not interfere with the ability of these cells to form a tumour when the node is transplanted. Thus, there was no evidence that nonspecific antigenic stimulation of the node altered its reactivity to isografted tumour cells.

8. *Comparison of the tumour cell content of nodes draining subcutaneous or intradermal tumours*

Unlike tumours from s.c. inocula, those from i.d. inocula grow to a moderate size without infiltration of the deep tissues; radical excision of i.d. tumours can therefore be accomplished with ease and efficiency. To prepare for experiments requiring tumour excision, it was necessary to establish that the frequencies of tumour formation by nodes from i.d. tumours and s.c. tumours were similar.

In 4 separate experiments in which regional nodes were transplanted 8–10 days after the i.d. injection of 25,000 tumour cells, when the average weight of the trimmed tumours was 130 mg, the pooled frequency of tumour formation by the nodes was 21/58 (36%). There were no significant differences between the frequencies in the separate experiments and no difference between the pooled value for the i.d. tumours and that for the control subcutaneous tumours, 47/117 (40%), given in Table III. Thus, the technical requirement of a change to i.d. tumours, as used in experiments to be described, was not associated with any change in the frequency of tumour formation from node transplants. This is in spite of the fact that i.d. tumours are frequently distinguished by central, dry ulceration.

9. *Comparative frequency of tumour formation by nodes isogeneically transplanted from tumour bearing mice and those left* in situ *in mice whose tumours were excised*

It was of interest to see whether the ~40% incidence of tumour formation from isogeneically transplanted nodes would be similarly expressed by nodes left *in situ* in equivalent mice whose survival had been prolonged by excision of their tumours.

Four separate experiments were carried out in which about 20 mice received equal i.d. inocula of about 20,000 tumour cells. In each experiment, on the 5th, 9th or 10th day after implantation (but on the same day in any one experiment), the mice were randomly distributed into 2 groups; mice of one group had their i.d. tumours radically excised and were observed for development of tumour growth in their regional lymph nodes for a period varying from 84 to 125 days in different experiments; mice of the other group were killed and their regional nodes were isogeneically transplanted.

The frequency of tumour formation by the transplanted nodes was 17/38 (45%). Of 43 mice which were kept under observation with their tumours excised and their nodes left intact, 5 developed local recurrence at the operation site 10–14 days after operation, and 7 developed pulmonary or mediastinal growths causing sickness or death between the 23rd and 58th days (mean 32) after operation; 2 of the mice with pulmonary metastasis had secondary disease elsewhere—one in the ovary, the other in the spinal column; none of the mice developing local recurrence or intrathoracic growth showed any sign of macroscopic growth in the node. Of the 31 remaining tumour-excised mice observed for at least 84 days, only one developed progressive growth in the axillary node; this was apparent 14 days after operation.

The pooled results from the 4 experiments thus showed that 17/38 (45%) of the transplanted nodes grew into tumours whereas only 1/31 (3%) of mice surviving free of intrathoracic disease or recurrence developed progressive tumour growth in the regional node. The difference between these incidences is highly significant ($P<0{\cdot}001$).

One possible interpretation of the above finding is that failure of tumours to form in the intact nodes of tumour-excised mice was due to restraint by immunological resistance induced in these mice by the previous growth of tumour in them; the normal mice to which nodes were transplanted had no such conditioning. The experiments described in the next section were done to test this immunologically orientated hypothesis.

10. *Comparative frequencies of tumour growth from regional nodes, either left* in situ *or autotransplanted, in mice whose tumours were excised*

In 3 separate experiments, tumours were excised from a number of mice which had grown i.d. tumours from an inoculum of 25,000–50,000 tumour cells given 7 or 8 days previously. The operated mice were randomly distributed to 2 groups; mice of one group were retained for observation without further interference; those of the other group had their regional nodes excised and *autotransplanted* to the opposite flank. The frequency of tumour growth from nodes autotransplanted or left *in situ* were respectively 8/25 (32%) and 1/24 (4%), their difference being significant ($0{\cdot}02>P>0{\cdot}01$). The incidence of growth from autotransplanted nodes (8/25–32%) is not significantly different from the pooled incidence of growth from nodes isotransplanted from mice bearing similar i.d. tumours, *i.e.* 38/96 (40%). Thus, the relative failure of intact regional nodes to give rise to tumours in tumour-excised mice is evidently not due to immunological activation by the previous growth of tumour in them.

11. *Latent periods for tumour formation from transplanted regional nodes*

The sites of transplantation of nodes were palpated every 2–3 days for detection of tumour initiation. Nodes themselves could be palpated for a few days after transplantation but it was possible to distinguish this transient palpability from the later onset of tumour growth, which was always progressive. Latent period (LP) data from the earliest node transplant experiments were excluded from analysis to allow for the development of observer experience and consistency.

The range of LPs for 76 tumour forming node transplants was 9–33 days, mean $18{\cdot}7\pm5{\cdot}0$ days. It is to be noted from the Figure that this mean value is very close to that expected for tumours arising from measured inocula of tumour cells at the $TD_{50}$ level of tumour takes (18 days). A further comparison was made, employing data from 5 recent subcutaneous assays of this tumour: 91 tumours which arose from mean cell numbers which were within $\pm0{\cdot}5$ log of the $TD_{50}$ values obtained in the assays to which they contributed had a mean LP of $17{\cdot}8\pm4{\cdot}1$ days. This value, again, is quite close to that for tumours arising from nodes.

It is clear that the mean LP for tumour initiation, as well as the frequency (30–50%) of tumour growth from transplanted regional nodes, is consistent with the hypothesis that all the nodes draining this tumour contain tumour cells, but the mean number of cells is close to the $TD_{50}$ level as determined by transplantation assays; that is, about 14 cells.

### DISCUSSION

The mechanism of metastasis is conveniently considered and investigated in terms of two phases and the influences upon them: firstly, the release of cells from the tumour as emboli in the blood or lymph vessels; and secondly, the seeding of such embolized cells in the organs to which they are carried. Our finding of a relatively high frequency ($\sim$40%) of tumour growth from auto- or isotransplanted regional nodes, contrasting with the rarity of tumour growth in equivalent nodes left *in situ*, leaves no doubt that our positive node transplants were revealing transnodal passage of tumour cells as distinct from incipient nodal metastasis.

Without the large background of data for the transplantation kinetics of this tumour, obtained from over 100 subcutaneous transplantation assays, it could be concluded from our $\sim$40% frequency of positive node transplants that tumour cells were either present or not present in the nodes at the time of transplantation, the case being decided by some intermittent influence on the tumour or node or some inhomogeneity of the donor or recipient mice. However, the overall frequency of tumour formation from the transplanted nodes, and the distribution of the latent periods for tumour formation, are consistent with a hypothesis that all the nodes contain a similar limited number of tumour cells. Reference to the Poisson distribution of tumour take frequency in relation to the log number of cells injected (Hewitt, Chan and Blake, 1967) indicates that only a two-fold increase of cell number is required to raise this frequency from 30% to 50% and that a ten-fold increase would raise the frequency to almost 100%. No such frequency was approached in any of our experiments. If, as might be supposed, the total number of cells reaching the regional node increases with some function of the tumour volume growth curve which integrates both cell population size and time, then at least a ten-fold increase in this function is represented by the data shown in Table II, although the take frequency of transplanted nodes increases by a factor of less than 2. We conclude that the nodal content of tumour cells is relatively insensitive to the time/size function of the tumour cell population drained and that the tumour cells reaching a node do not accumulate in it.

Following the above considerations, we interpret our evidence as follows: during tumour growth a continuous stream of tumour cells leaves the tumour and passes *via* the lymphatics and node to reach one of the main terminal lymphatic trunks, the peripheral sinuses of the node merely forming a segment of this free channel and exerting no more restraint upon the flow than the lymphatics leading to and from it; the small number of tumour cells in the node at the time of its excision and transplantation can be regarded as a " cut " from this channel of flow. Certainly, the number of cells leaving a tumour *via* the lymphatics in unit time would be expected to increase with tumour size; but so also would the volume of lymph, so that the *density* of tumour cells in the lymph could remain fairly constant; the larger exodus of tumour cells from larger tumours would be accommodated by an increased rate of lymph flow. In this situation, the " cut " from the lymph flow channel represented by the excised node would not be expected to have a tumour cell content which was closely correlated with tumour size, provided there is no great increase in the volume of the peripheral sinuses. Since this interpretation is based on the reasonable assumption that the lymph flow rate is proportionate to the volume of tissue drained, it would be expected to apply to any tumor in which transnodal passage of tumour cells could be similarly demonstrated.

The resumption of lymph flow following radical excision of a tumour would be expected to flush out, in due time, any tumour cells in the node at that time. The question arises: what is the fate of the large number of tumour cells which pass through the node during the residence of a tumour? Anatomy prescribes that they would reach the venous blood, where their status would be similar to that of intravenously injected cells, and we have shown that over 99% of the clonogenic cells so injected are evidently destroyed within a few hours of their reaching the lungs (see Subsection 2 of Results). Thus, although we have not elucidated the mechanism responsible for this massive destruction of cells, it would seem that a single mechanism is involved in the destruction of intravenously injected and transnodally passaged cells.

Since the current surge of interest in anti-tumour immunity has, in our view, encouraged uncritical attachment of immunological interpretations to various phenomena encountered in the behaviour of clinical and experimental cancer, we feel it to be necessary to detail our reasons for excluding such interpretation of the experiments described here. This is specially so because our experiments have involved an immunologically significant organ in which clonogenic cells can be demonstrated but which rarely give rise to tumour growth when left *in situ*.

1. The tumour arose spontaneously in a mouse of the same inbred colony as that providing the transplant recipients; the antigenicity usually associated with chemically induced or allografted tumours can therefore be excluded.
2. The transplantation kinetics of the tumour (Porter *et al.*, 1973) reveals no evidence of heterogeneity of the mice in respect of their acceptance of small numbers of tumour cells.
3. Not a single failure to take has been observed in over 600 mice used for maintenance of the tumour.
4. The $TD_{50}$ is small and cannot be increased by prior treatment of the mice with lethally irradiated cells.
5. The production of tumours by isogeneically transplanted nodes from tumour bearing mice entails the conditions required for *adoption* by the recipient of any immunity generated in the donor (Mitchison, 1953), yet no such immunity was manifested.
6. There was no difference of tumour incidence or latent period between nodes autotransplanted to mice conditioned by tumour growth and nodes isotransplanted to mice with no previous experience of the tumour.
7. The destruction of tumour cells in the lung as demonstrated by our transplantation of lungs from i.v. injected mice, occurs within a few hours; this is too short a time for the induction of a rejection response in an unconditioned host.

Our failure to increase the incidence of tumour formation from regional node transplants by incision, massage or injection of the primary tumour, or by the coincident adjacent growth of allografted tumour, suggests that the increased functional demand put upon the node by such procedures did not increase the liability of a node to seed and grow the tumour cells reaching it. This evidence does not favour the hypothesis that induction of frank metastasis in a node may follow depletion of its functional capacity to " resist " tumour growth.

The question whether our findings for this system have general significance for others depends on whether embolization of tumour cells in lymph is a common characteristic of tumours of different types. We should mention in this context that we have found that the number of tumour cells required to initiate tumours after their subcutaneous injection, as given by the $TD_{50}$, varies very widely (from 1 to 10,000) between different tumours. These differences imply large differences in the efficiency of whole node transplantation as a test for the presence of small numbers of tumour cells passing through the node.

Transnodal passage of tumour cells has been demonstrated by Fisher and Fisher (1966, 1967) and by Madden and Gyure (1968). In both cases tumours known to be antigenic were used and the tumour cells were injected in very large numbers directly into the afferent lymphatics. These experiments disposed of the classic notion that lymph nodes may act as efficient barriers to the dissemination of tumour cells. However, we are not aware of any previous report of the continual transnodal passage of tumour cells naturally disseminated from a non-antigenic tumour of a common clinical type; a significant feature of our experiments was that the demonstration involved no anaesthesia, surgical interference or disturbance of the hydrodynamics of lymph flow.

Whether *embolization* or *seeding* of tumour cells is the predominant issue deciding the establishment of metastatic growth in organs has been an important consideration in the study of dissemination *via* the blood, which topic has been much more intensely investigated. Greene and Harvey (1964) were able to divide the hamster tumours they examined into two classes: those in which tumour cells were widely distributed in organs which failed to develop metastases and those in which metastases commonly arose in organs in which cells could be demonstrated by transplantation. In the case of lymphatic dissemination of the tumour used here, it is clear that the occurrence of embolization is not at all the critical factor deciding nodal metastasis. Our current studies are therefore directed to an examination of possible influences on the seeding and growth of tumour cells passing through the node.

We believe that transnodal passage of tumour cells has first to be quantitated, as here, before adequate appraisal can be made in any system of the relative importance of dissemination and seeding in the establishment of progressive nodal metastasis

We are grateful to Dr L. J. Peters of this Laboratory for having taught us the technique used for intradermal injection of mice, and to Miss Angela Walder, A.I.A.T., for the breeding and care of all the mice used. The cost of the investigation was met exclusively by the Cancer Research Campaign.

### REFERENCES

Fisher, B. & Fisher, E. R. (1966) Transmigration of Lymph Nodes by Tumor Cells. *Science, N.Y.*, **152**, 1397.

Fisher, B. & Fisher, E. R. (1967) Barrier Function of Lymph Node to Tumor Cells and Erythrocytes. I. Normal Nodes. *Cancer, N.Y.*, **20**, 1907.

Greene, H. S. N. & Harvey, E. K. (1964) The Relationship between the Dissemination of Tumor Cells and the Distribution of Metastases. *Cancer Res.*, **24**, 799.

Hewitt, H. B. (1956) The Quantitative Transplantation of Sarcoma 37 into Subcutaneous Air Pouches in Mice. *Br. J. Cancer*, **10**, 564.

Hewitt, H. B. (1966) The Effect on Cell Survival of Inhalation of Oxygen under High Pressure during Irradiation *in vivo* of a Solid Mouse Sarcoma. *Br. J. Radiol.*, **39**, 19.

Hewitt, H. B., Chan, D. P. & Blake, E. (1967) Survival Curves for Clonogenic Cells of a Murine Keratinising Squamous Carcinoma Irradiated *in vivo* or under Hypoxic Conditions. *Int. J. radiat. Biol.*, **12**, 535.

Hummel, K. P., Richardson, F. L. & Fekete, L. (1966) In *Biology of the Laboratory Mouse*. Ed. E. L. Green. London: McGraw-Hill. p. 247.

Klein, G. (1970) Immunological Factors Affecting Tumour Growth. *Br. med. J.*, iv, 418.

Lindberg, R. (1972) Distribution of Cervical Lymph Node Metastases from Squamous Cell Carcinoma of the Upper Respiratory and Digestive Tracts. *Cancer, N.Y.*, **29**, 1446.

Madden, R. E. & Gyure, L. (1968) Translymphnodal Passage of Tumour Cells. *Oncology*, **22**, 281.

Marchant, J. (1968) Antigenic Properties of Spontaneously-occurring Tumours of Mice. In *46th Annual Report of the British Empire Cancer Campaign*. London. p. 250.

Mitchison, N. A. (1953) Passive Transfer of Transplantation Immunity. *Nature, Lond.*, **171**, 267.

Porter, E. H., Hewitt, H. B. & Blake, E. R. (1973) The Transplantation Kinetics of Tumour Cells. *Br. J. Cancer*, **27**, 55.

Prehn, R. T. & Main, J. M. (1957) Immunity to Methylcholanthrene-induced Sarcomas. *J. natn. Cancer Inst.*, **18**, 769.


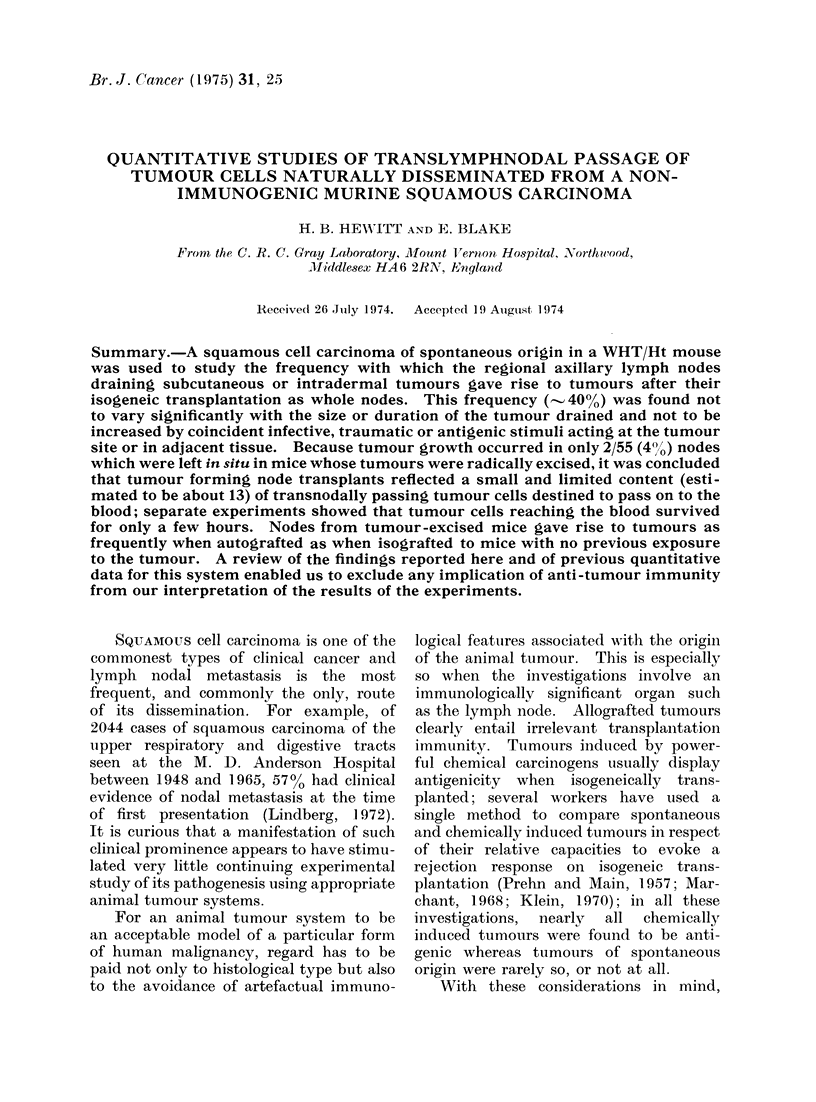

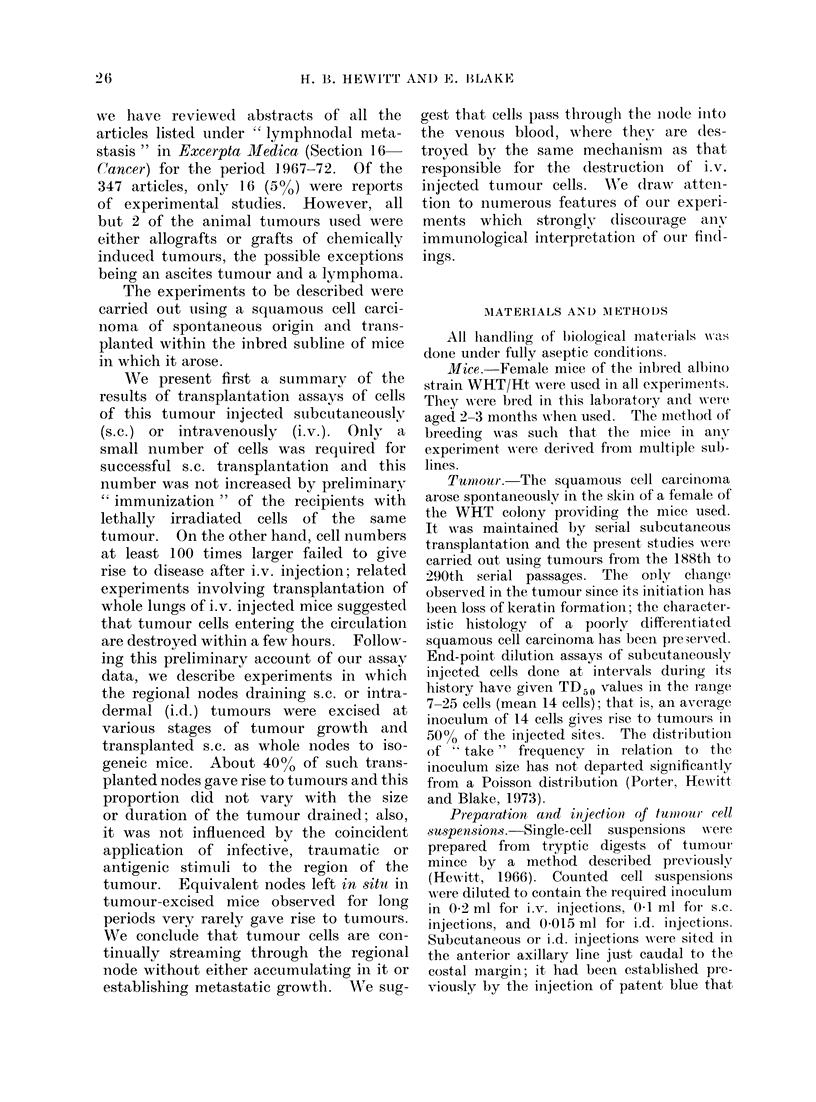

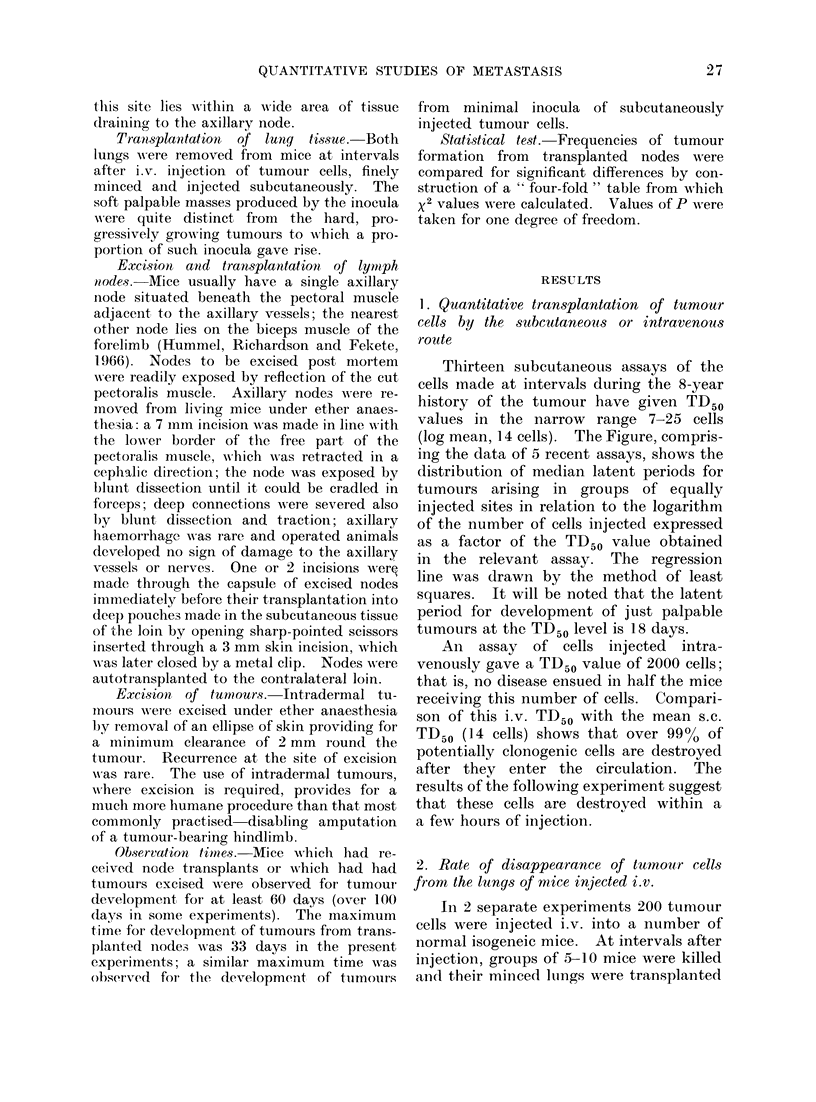

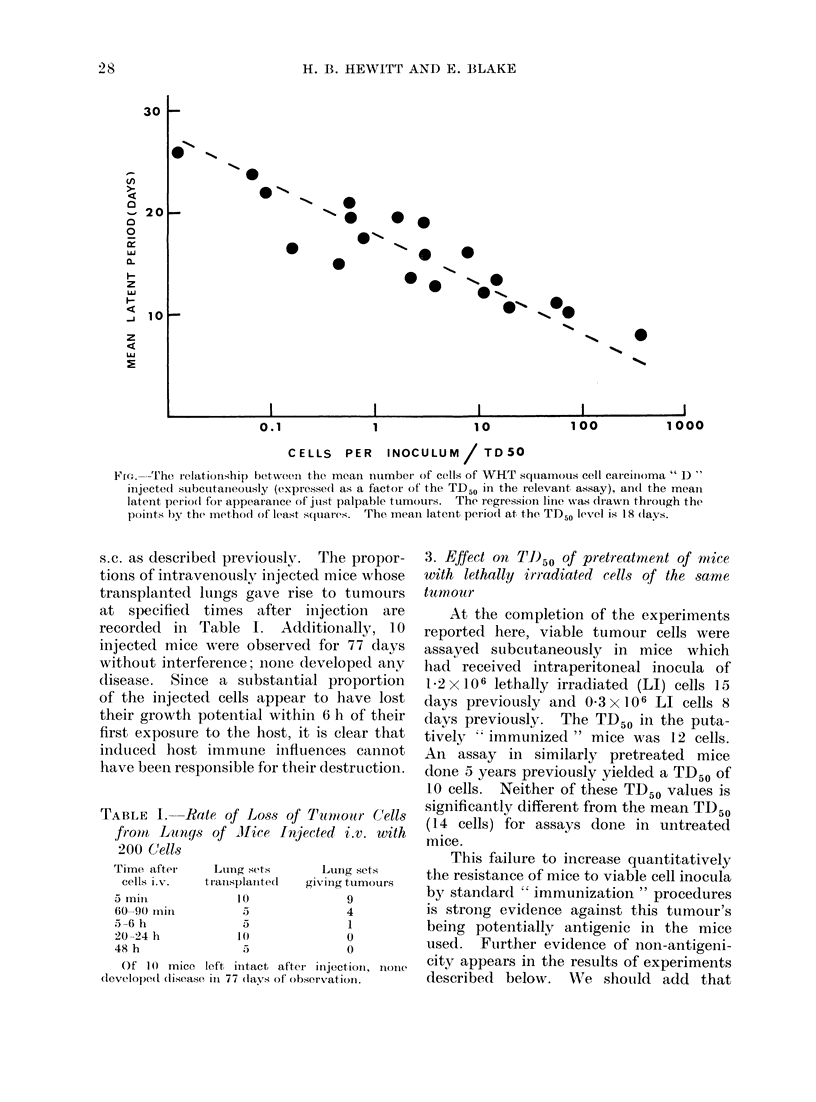

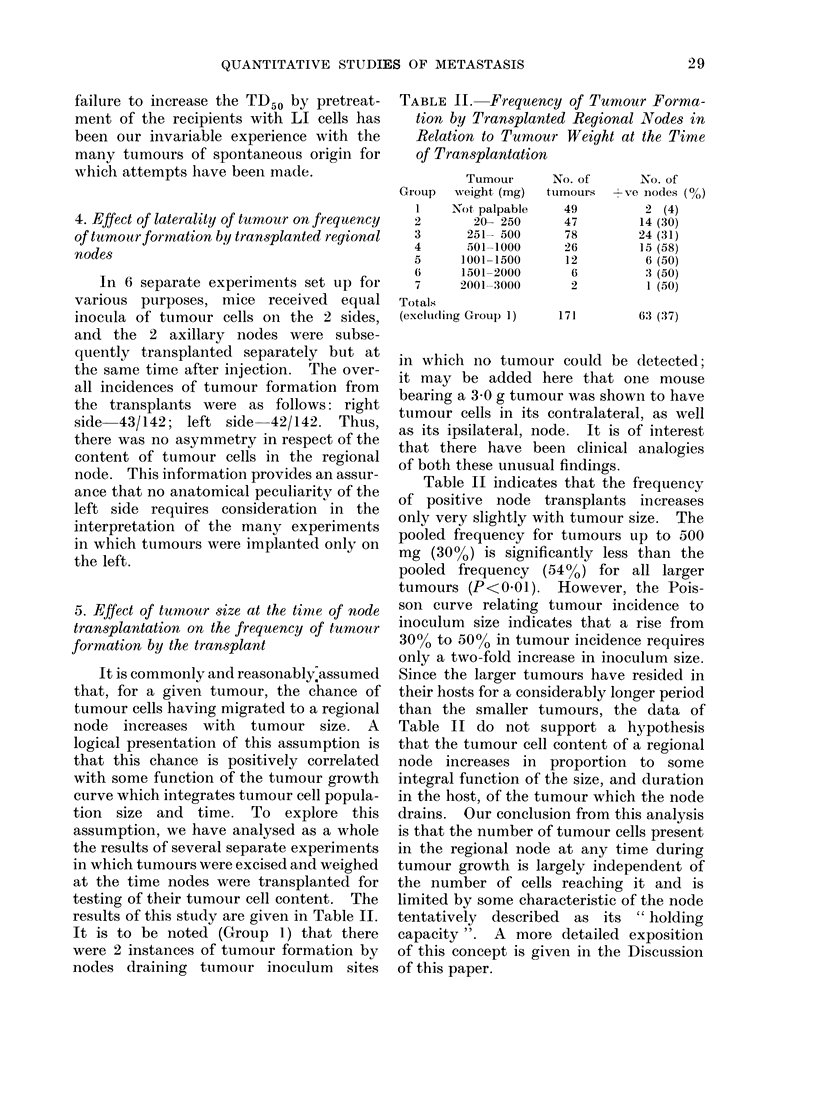

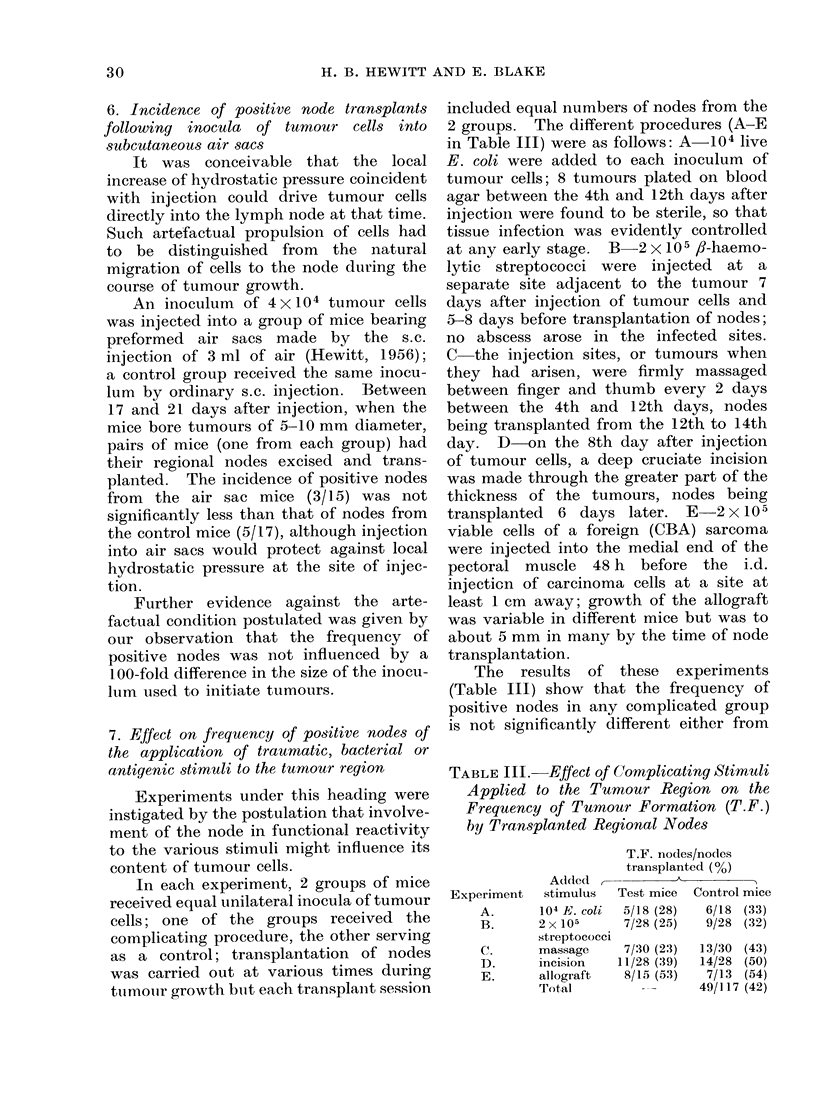

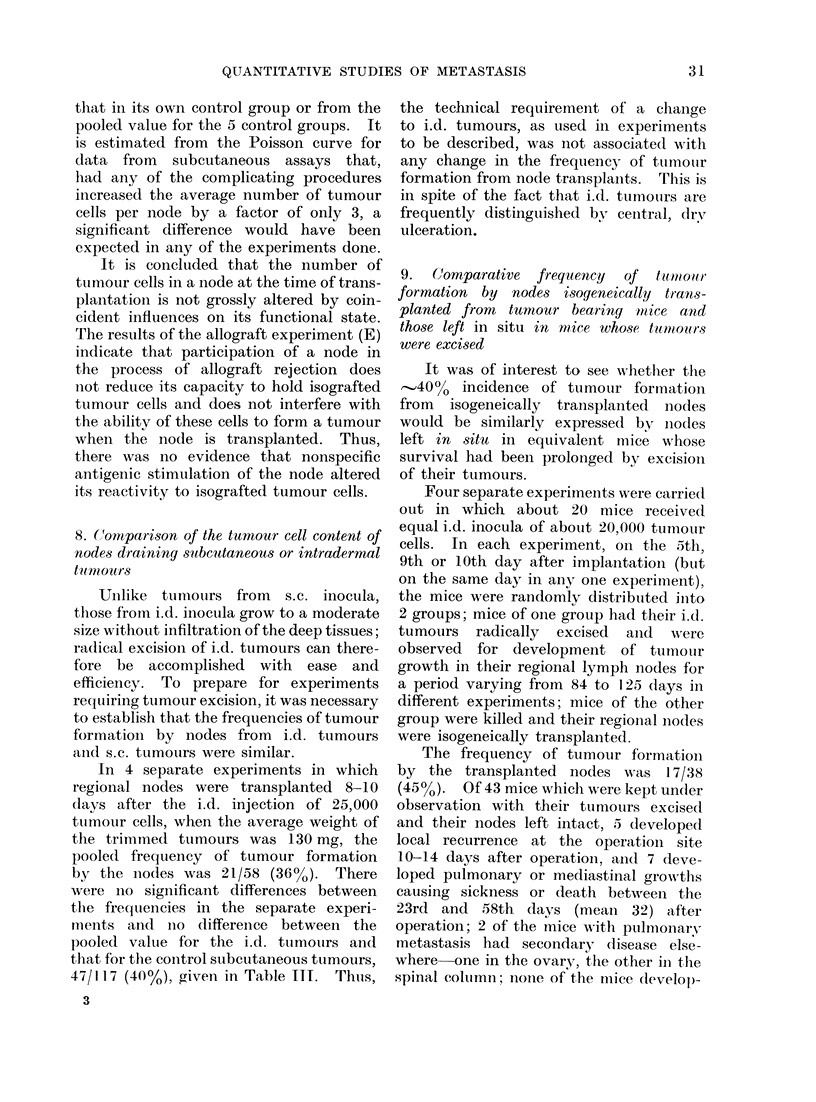

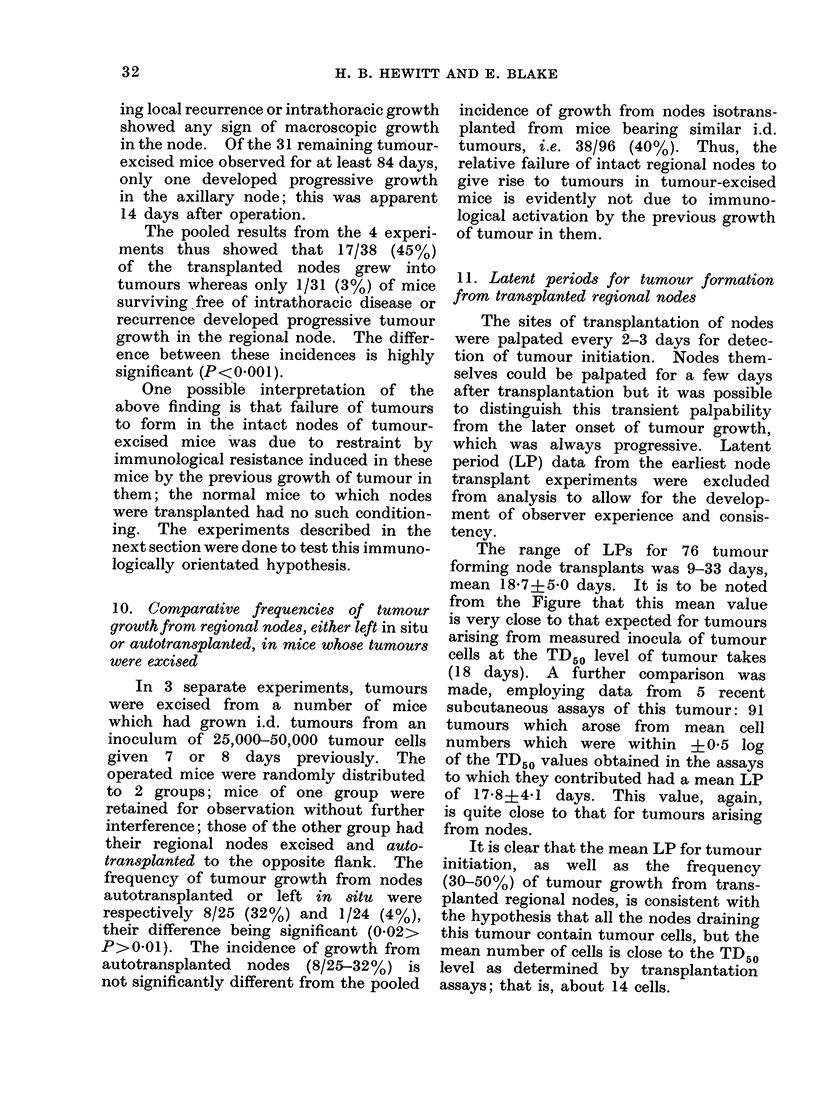

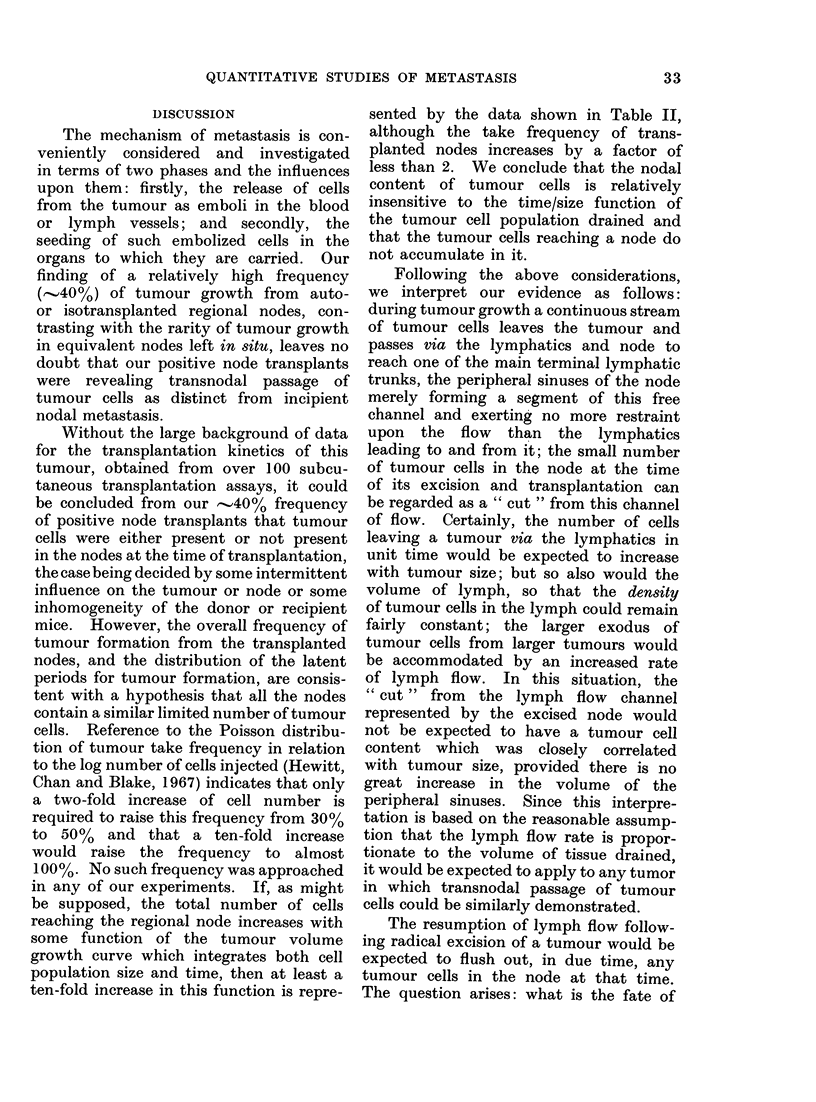

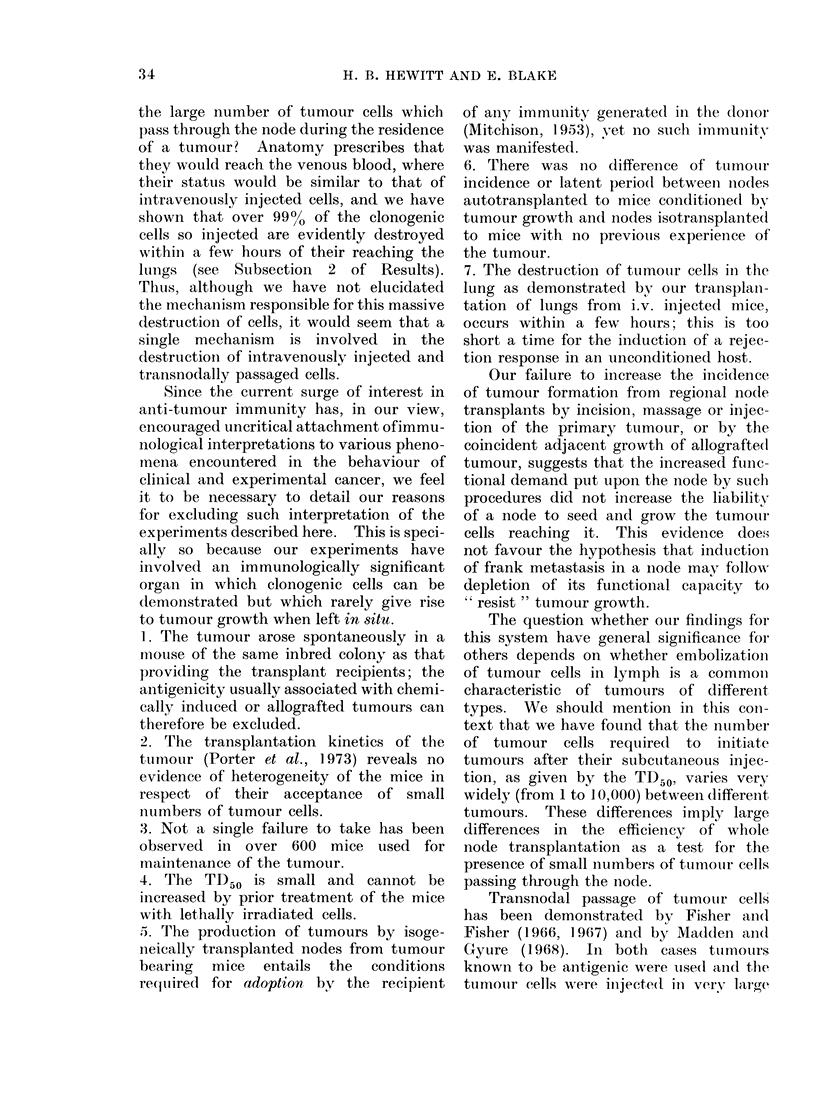

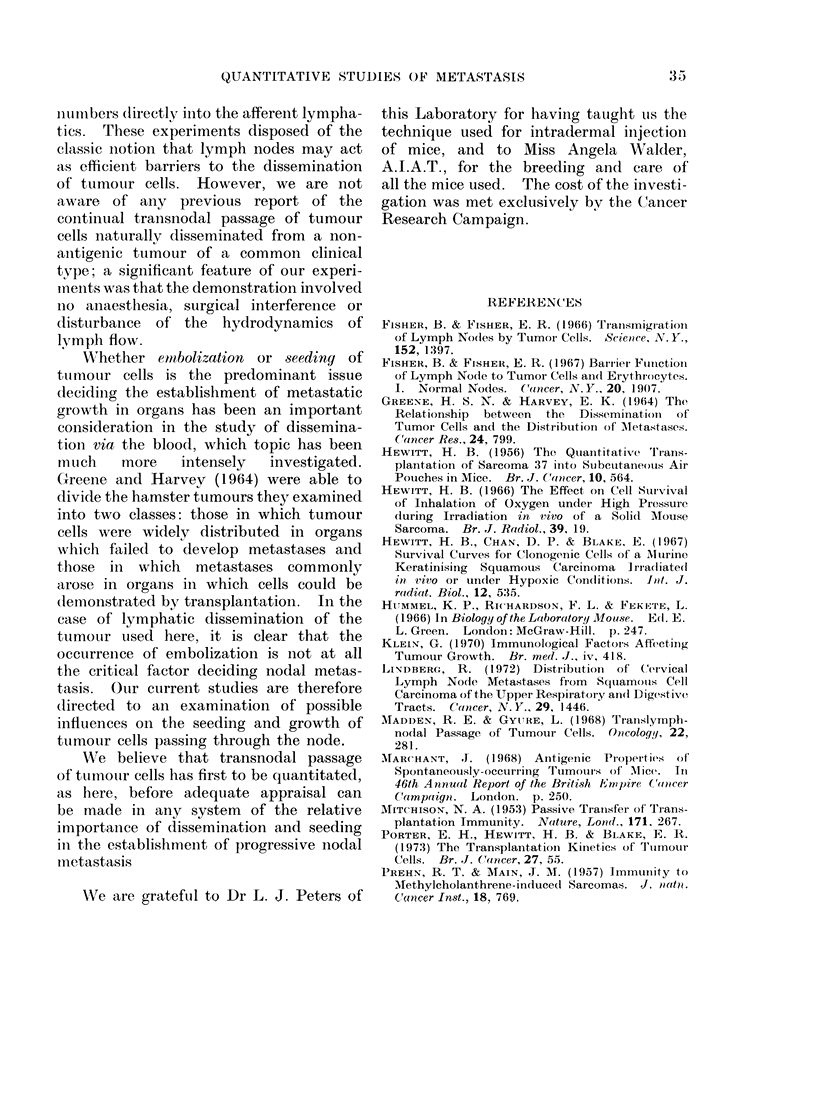


## References

[OCR_01237] Fisher B., Fisher E. R. (1966). Transmigration of lymph nodes by tumor cells.. Science.

[OCR_01247] GREENE H. S., HARVEY E. K. (1964). THE RELATIONSHIP BETWEEN THE DISSEMINATION OF TUMOR CELLS AND THE DISTRIBUTION OF METASTASES.. Cancer Res.

[OCR_01253] HEWITT H. B. (1956). The quantitative transplantation of sarcoma 37 into subcutaneous air pouches in mice.. Br J Cancer.

[OCR_01258] Hewitt H. B. (1966). The effect on cell survival of inhalation of oxygen under high pressure during irradiation in vivo of a solid mouse sarcoma.. Br J Radiol.

[OCR_01276] Klein G. (1970). Immunological factors affecting tumour growth.. Br Med J.

[OCR_01280] Lindberg R. (1972). Distribution of cervical lymph node metastases from squamous cell carcinoma of the upper respiratory and digestive tracts.. Cancer.

[OCR_01297] MITCHISON N. A. (1953). Passive transfer of transplantation immunity.. Nature.

[OCR_01306] PREHN R. T., MAIN J. M. (1957). Immunity to methylcholanthrene-induced sarcomas.. J Natl Cancer Inst.

